# What barriers need to be removed to incorporate resistance exercise into the lives of older adults with long‐term diabetes and prefrailty? A deductive framework analysis using the Capability, Opportunity, Motivation and Behaviour (COM‐B) behavioural science model

**DOI:** 10.1111/dme.70367

**Published:** 2026-05-16

**Authors:** Rachel Stocker, Siân Russell, Guy S. Taylor, Miles D. Witham, James A. M. Shaw, Daniel J. West

**Affiliations:** ^1^ School of Biomedical, Nutritional and Sport Sciences Newcastle University Newcastle upon Tyne UK; ^2^ School of Natural and Environmental Sciences Newcastle University Newcastle upon Tyne UK; ^3^ Population Health Sciences Institute Newcastle University Newcastle upon Tyne UK; ^4^ NIHR Newcastle Biomedical Research Centre Newcastle upon Tyne Hospitals NHS Foundation Trust, Cumbria, Northumberland, Tyne and Wear NHS Foundation Trust and Newcastle University Newcastle upon Tyne UK; ^5^ Translational and Clinical Research Institute Newcastle University Newcastle upon Tyne UK

**Keywords:** aged, diabetes mellitus, type 1, diabetes mellitus, type 2, frailty, hypoglycaemia, qualitative research, resistance training

## Abstract

**Aims:**

Maintaining physical function, and thus independence, is essential for people ageing with diabetes. For older adults with long‐term insulin‐treated diabetes, managing frailty, preserving body mass index (BMI) and muscle strength and mitigating hypoglycaemia risk are key challenges. Resistance training (RT) offers benefits, including reduced hypoglycaemia risk, increased bone density and improved muscle strength, yet its uptake remains low. This study explores behavioural influences and barriers to RT participation among older adults living with insulin‐treated diabetes and prefrailty.

**Methods:**

A qualitative approach was employed, involving semi‐structured interviews with individuals living with prefrailty aged 60 years or older with insulin‐treated diabetes (type 1 diabetes (*n* = 12); type 2 diabetes with BMI < =30 kg/m2 (*n* = 4)). Frailty was assessed using the Rockwood Clinical Frailty Scale (3–4). Data were analysed using framework analysis and aligned to the COM‐B model of behaviour change to deductively identify barriers and facilitators.

**Results:**

Barriers to RT were identified across psychological capability, physical capability and social opportunity COM‐B domains. Key barriers included fears of fatigue, hypoglycaemia and injury, diabetes‐related complications and difficulties using RT equipment. Outdated advice about exercise safety and lack of awareness of RT's benefits further hindered participation. Facilitators included tailored education on diabetes‐specific RT benefits, a supportive, flexible training environment and the presence of an exercise‐competent partner.

**Conclusions:**

This study highlights perceptual and practical barriers that discourage older adults with diabetes and prefrailty from engaging in resistance exercise. Addressing these barriers through educational initiatives and creating adaptable exercise programmes could enhance exercise participation rates in this population.

**What's New?**

- Resistance training benefits older adults with insulin‐treated diabetes, but uptake is low and perceived risks are common.
- In interviews with 16 older adults with insulin‐treated diabetes and prefrailty, barriers clustered around capability (fatigue, complications, equipment skills) and social opportunity (support, training environment), with fear of hypoglycaemia and injury prominent.
- Programmes should include diabetes‐specific resistance training education, skills training with supervision and practical social support.

## 1 INTRODUCTION

One in five people aged over 65 years has diagnosed diabetes, and this trend is rising with an ageing population.^1^ In those with long‐term type 1 diabetes and type 2 diabetes requiring insulin treatment, disease management and ageing carry additional complexities, such as more frequent hypoglycaemia, impaired hypoglycaemia awareness and potentially severe events requiring assistance from others; variable appetite; sarcopenia (progressive loss of muscle mass and strength associated with ageing); and challenges with optimal insulin dose management.^2^, ^3^

Frailty and sarcopenia are prevalent among older adults, and diabetes may compound these conditions. Mechanisms, such as hypoglycaemia and increased insulin sensitivity, are associated with reduced muscle mass and strength.^4^, ^5^ The prevalence of frailty among older adults with diabetes is significantly higher—between 32% and 48%—compared to the 5%–10% observed in older adults without diabetes.^6^, ^7^ This higher prevalence contributes to increased risks of falls, disability, osteoporosis, fractures and mortality.^8^, ^9^ These risks are particularly impactful for those on long‐term insulin therapy, potentially leading to worse hypoglycaemia and further deterioration in muscle health. Maintaining physical function and combating frailty is key to this population's overall wellbeing.

While aerobic exercise, such as walking, is often recommended for diabetes management, it carries an increased risk of hypoglycaemia^10^ particularly for older adults who may already have reduced hypoglycaemia awareness or blood glucose instability. This risk complicates the promotion of aerobic activity in this group, as the likelihood of dangerous hypoglycaemic events grows with age.^11^ For those living with frailty, calorie‐burning aerobic exercise may present additional challenges to maintaining a healthy BMI and muscle mass. Frailty is characterised by reduced physiological reserve and muscle loss, meaning that exercises which increase energy expenditure without sufficient caloric or protein intake can exacerbate catabolism. In this context, aerobic activity may contribute to further reductions in lean mass and strength.

Resistance training (RT), defined as short intense muscular contractions conducted in sets and repetitions against a fixed load, is recommended as a first‐line treatment for sarcopenia and frailty because it can improve muscle strength and power, muscle mass and physical performance,^12^ and it has been shown to increase bone density and reduce falls in older adults.^13^, ^14^ For those living with diabetes, RT typically involves fewer blood glucose fluctuations than aerobic exercise due to a different hormonal response, including increased production of anti‐hypoglycaemic hormones including adrenaline and cortisol.^10^ This metabolic milieu suggests that RT may be a safe and effective therapeutic approach for older adults with insulin‐treated diabetes.

Despite the clear benefits of regular exercise (including RT), uptake among older adults remains low; for example, a large UK accelerometer‐based study found only ~10%–15% achieved guideline activity levels,^15^ and a recent global study reports similarly low participation in physical activity in older populations.^16^ Most adults with diabetes do not achieve recommended aerobic activity volumes, and typical free‐living moderate‐to‐vigorous physical activity levels appear low, with a sizeable subgroup doing near‐zero moderate‐to‐vigorous physical activity on many days, especially in older cohorts.^17^, ^18^

Although RT is generally considered safe for this population and appears to be a promising therapeutic addition to diabetes management, the specific barriers and facilitators to this group's participation remain unclear. Identifying these factors is important for developing tailored interventions to help this vulnerable group engage in RT safely, to improve their overall health, and reduce their risk of frailty and sarcopenia.

One approach to understanding these factors is the Capability, Opportunity, and Motivation Model of Behaviour (COM‐B).^19^ This behaviour science framework, well‐evidenced and widely used in health research, suggests that behaviour (B) is influenced by three components: capability (C), opportunity (O) and motivation (M). Capability refers to an individual's psychological and physical ability to perform a behaviour, including necessary skills and knowledge. Opportunity involves external factors that enable behaviour, such as social or environmental resources. Motivation includes both reflective (deliberate decisions) and automatic (habits or impulses) processes.^19^ These concepts are summarised in Table 1.

**TABLE 1:** The COM‐B model components and associated definitions.

COM‐B component	Description
**Capability**	**The person must be able to perform the behaviour**
Physical capability	They must have the physical skill, strength or stamina.
Psychological capability	They must have the relevant knowledge or psychological skills, strength or stamina.
**Opportunity**	**There must be the opportunity to perform the behaviour**
Physical opportunity	There should be the necessary time and resources as well as an accessible location. Aspects of the physical environment may also facilitate or cue the behaviour.
Social opportunity	The behaviour should be socially acceptable. Aspects of the social environment may also facilitate or cue the behaviour.
**Motivation**	**The person should be motivated to perform the behaviour**
Reflective motivation	Refers to motivation that involves plans and evaluations.
Automatic motivation	Refers to motivation that involves automatic processes such as emotional reactions, desires, impulses, inhibitions, drive states and reflex responses.

The COM‐B model has been used extensively to understand and predict health behaviours, including in diabetes.^20^ In the context of RT for older adults with diabetes, understanding how these elements interact is important for developing interventions that enhance capability (e.g., education), opportunity (e.g., accessible environments) and motivation (e.g., encouraging positive attitudes and increased self‐efficacy).

### 1.1 Study aim and objectives

This qualitative descriptive study explores the barriers and facilitators of RT in older adults living with insulin‐treated diabetes and prefrailty, using the Capability, Opportunity, Motivation and Behaviour (COM‐B) model. By understanding these factors, we can develop targeted interventions to increase the uptake and maintenance of RT in this vulnerable population.

## 2 METHODS

### 2.1 Design

A qualitative descriptive study design was used as the aims of this study were exploratory. Semi‐structured interviews were conducted with participants between June 2021 and August 2022. This study received ethics approval from the North East—Newcastle & North Tyneside 2 Research Ethics Committee (ref: 20/NE/0178). This study was originally nested within the EXercise to Prevent frailty and Loss Of independence in insulin treated older people with DiabetEs (EXPLODE) pilot randomised controlled trial (ISRCTN13193281); however, the trial was severely disrupted by the COVID‐19 pandemic and did not proceed to completion. The qualitative interview study reported here was the only component conducted.

### 2.2 Participants and recruitment

A purposive sampling strategy was used to recruit participants to this study. Participants were recruited via poster advertisements and screening in diabetes clinics, through adverts placed on the Juvenile Diabetes Research Foundation and Diabetes UK newsletters, and through social media. Inclusion criteria were: a diagnosis of either type 1 diabetes or type 2 diabetes treated with exogenous insulin; aged 60 years or older; living with prefrailty (assessed by the screening clinician or the research team using a Rockwood Clinical Frailty Score of 3 or 4^21^, ^22^); with no history of severe cardiovascular or renal health issues within the preceding 12 months; and residing in the United Kingdom. Those with type 2 diabetes were also required to have a body mass index (BMI) of <30 kg/m^2^. This criterion was used to reduce heterogeneity and limit confounding from obesity‐related differences in physical function, body composition and frailty phenotype, which may independently influence resistance training participation in older adults with insulin‐treated type 2 diabetes. It also reflected our clinical observations, particularly in secondary care, that some older adults with insulin‐treated type 2 diabetes and a BMI <30 kg/m^2^ experience issues similar to those seen in older adults with type 1 diabetes at this stage of disease. These include frailty, the fact that further weight loss may be undesirable, and the need to manage insulin dosing and hypoglycaemia risk. The intention was therefore not to focus on diabetes type itself, but to recruit a more clinically comparable subgroup for this exploratory qualitative study.

The study did not predefine a sample size, reflecting its adaptive methodology that concluded recruitment when the study had reached data and analytical sufficiency,^23^ as identified by the research team. Data sufficiency can be defined as the point in data collection and analysis where it is deemed that sufficient data have been collected to meaningfully answer the research question(s). We anticipated that we would recruit fewer individuals with type 2 diabetes on insulin who also met the BMI criteria, further restricting the pool of potential participants. This was evident in our recruitment, despite extra targeted efforts by those working in a specialist secondary care clinic focused on insulin‐treated type 2 diabetes.

### 2.3 Procedure

Potential participants were screened to check they met the inclusion criteria, in particular, the Rockwood Clinical Frailty Score assessment. Informed consent was obtained verbally and audio recorded for all participants; this was preferred and approved by the UK Health Research Authority over written informed consent due to the low‐risk, remote and geographically wide nature of the study.

Interviews were conducted by RS, a member of the research team, over the telephone. No non‐participants were present during the interviews. RS is an experienced qualitative research academic with a background in psychology and research experience in diabetes. The research team did not have any prior contact or relationship with any participants. Interviews ranged in duration from 00:20:00 to 01:17:00 (mean ± SD, 40.0 ± 13.5 min). All interviews were one‐off. All interviews were audio recorded, transcribed verbatim by a University‐approved transcription service, then anonymised by the study team. Transcripts were not returned to participants for comment or correction. Interviews followed a semi‐structured topic guide, which was developed by the study team using previous literature. Interviews focused on participants' history of diabetes, their current perceived health status, changes in diabetes management as they have aged, any diabetes‐related complications and the impact of these on their life, attitudes towards exercising and resistance training, and barriers and facilitators to resistance training. For the purposes of this paper, analysis focused deductively on the latter topics of the interviews. Field notes were taken post‐interview and used to aid contextual understanding and analysis.

### 2.4 Data analysis

Interview data were analysed deductively using framework analysis, with the COM‐B model used as the a priori analytic framework, by RS and SR. All interviews were imported into NVivo 14 software to support data management. Following an initial phase of data familiarisation, barriers and facilitators to RT were coded deductively, line‐by‐line to a framework of the six sub‐components of the COM‐B model (physical/psychological capability, social/physical opportunity and reflective/automatic motivation). Each sub‐component code was split into barrier and facilitator, ultimately generating 12 coding domains in the framework. The analysis was focused on identifying barriers and facilitators to resistance training participation and mapping these onto the COM‐B domains; no separate inductive analysis was undertaken. Differences in coding within domains were discussed between the research team (RS, SR and DW) on multiple occasions, and amended if necessary. Once completed, each coded domain was reviewed as a whole to understand collective barriers and facilitators.

### 2.5 Reflexivity and positionality

RS (female in her 30s) acted as the analysis lead. RS is a qualitative researcher in her 30s; RS has worked in diabetes‐related research, in collaboration with DW, for a number of years. She is a chartered psychologist with expertise in health psychology and in the application of the COM‐B model and other theories and frameworks of behaviour change.

RS also has a close relative who has lived with type 1 diabetes for over 20 years. This has shaped her long‐standing interest in the topic and may have influenced her interpretation of participants' accounts. To acknowledge and reflect on this potential influence during the research process, RS engaged in regular discussions considering the interview topic guide, recruitment procedures, participant communication, and developing analysis with DW and SR throughout the study.

DW (male in his 40s) has a PhD in clinical exercise physiology. His research focuses on exercise and nutrition in relation to chronic disease management, with a particular emphasis on diabetes. DW's wealth of knowledge supported RS's personal experiences from having an undue bearing on the analysis. SR (female in her 40s) has a PhD in medical sociology and is an experienced qualitative researcher. Her prior research has focused on health, illness and ageing, but without a focus on diabetes, adding an educated but non‐diabetes‐specific voice to the development of analysis.

Although this qualitative work was originally intended to sit alongside a wider trial, the RCT did not proceed because of restrictions related to the COVID‐19 pandemic. As a result, only the qualitative interview study reported here was conducted. Participants were aware at recruitment that they were taking part in interviews only, rather than being enrolled into the originally planned RCT.

## 3 RESULTS

Twenty‐five potential participants were screened, of which 16 met the criteria, and all 16 agreed to take part. This sample size consisted of 12 participants with type 1 diabetes and four participants with type 2 diabetes. Participant characteristics are listed in Table 2. All lived in the United Kingdom.

**TABLE 2:** Participant characteristics, overall and by diabetes type.

Characteristic	Total (*n* = 16)	Type 1 diabetes (*n* = 12)	Type 2 diabetes (*n* = 4)
Age, years	67.6 ± 8.0 (60–89)	68.5 ± 8.7 (60–89)	64.8 ± 5.7 (60–73)
Women, *n* (%)	9 (56.3)	8 (66.7)	1 (25.0)
Diabetes duration, years	29.8 ± 17.4 (10–70)	33.1 ± 18.5 (10–70)	19.8 ± 8.7 (12–31)
BMI, kg/m^2^	27.8 ± 4.1 (22–36)	28.3 ± 4.5 (22–36)	26.3 ± 2.8 (23–29)
Rockwood Clinical Frailty Scale score, *n* (%)			
3 (managing well)	7 (43.8)	5 (41.7)	2 (50.0)
4 (vulnerable)	9 (56.3)	7 (58.3)	2 (50.0)

*Note*: Data are mean ± SD (range) unless otherwise indicated. Rockwood Clinical Frailty Scale definitions: 3, “managing well” (medical problems well controlled; not regularly active beyond routine walking); 4, “vulnerable” (not dependent on others but symptoms limit activities; often feels “slowed up”).

Abbreviation: BMI, body mass index.

A summary of the results aligned to COM‐B domains can be found in Table 3. Domains with supporting quotations from participants can be found in Table 4. Barriers to resistance training were identified across all COM‐B domains and were most commonly located in the physical and psychological capability‐ and social opportunity‐related domains. Results from all COM‐B domains are presented in more detail below.

**TABLE 3:** Barriers and facilitators to resistance training reported by participants aligned to COM‐B domains.

COM‐B domain	Component	Barriers to RT	Facilitators to RT
Capability	Physical	Feeling slowed down with aging	Nil
		Diabetes management feeling harder with age	
		Specific physical limitations to training	
Capability	Psychological	Variable knowledge of managing diabetes, especially hypoglycaemia, during RT	Enablement to develop RT exercise skills
		Variable knowledge of the potential benefits of RT for diabetes	
		Lacking knowledge of how to use RT‐related equipment	
Motivation	Automatic	RT feels boring	Feeling supported by others to enable exercise
		Fear of injury	‘I will not let diabetes limit my choices’
		Fear of hypoglycaemia	
Motivation	Reflective	Having other exercise routines that do not involve RT	Use of CGM to monitor diabetes control during exercise
			Valuing improvements in health brought about by RT
Opportunity	Social	Feeling ‘too old’ to try a new exercise modality	Knowledgeable personal trainers to support RT for those with insulin‐treated diabetes
		Poor awareness in society and amongst personal trainers	
		Negative self‐comparison between themselves and regular gym‐goers	
		Being encouraged to limit exercise at diagnosis	
		Partner cannot/will not accompany them to exercise	
Opportunity	Physical	Financial cost of attending a gym	Nil
		Lack of time to attend a gym	

Abbreviations: BMI, body mass index; CGM, continuous glucose monitoring; RT, resistance training.

**TABLE 4:** COM‐B domains with supporting quotations from participants.

COM‐B Domain	Component	Code	Supporting quotations
Capability	Physical	***Barriers***	
		Feeling slowed down with aging	*“It's not being able to do as much as I want to do because obviously, you're slowing down.”* (Participant 5, woman, age 67, type 1 diabetes)
		Diabetes management feeling harder with age	*“It all just seems to be getting worse the older I get. It's one thing after another.”* (Participant 3, woman, age 63, type 1 diabetes)
			*“When you've had diabetes for so long it doesn't always follow the rules.”* (Participant 8, man, age 65, type 1 diabetes)
		Specific physical limitations to training	*“If the shoes aren't right then that limits how I can exercise and my mobility and everything else and that has been a huge challenge*… *If I can't balance and stand in my shoes then doing weights for anything else is difficult for me.”* (Participant 1, woman, age 80, type 1 diabetes)
		***Facilitators***	(nil)
Capability	Psychological	***Barriers***	
		Variable knowledge of managing diabetes, especially hypoglycaemia, during RT	*“I don't think you need to go pull weights at the gym because what that was doing for me when I was doing it as diabetic was just sending me very low and then I was correcting it and going very high*… *so yes in a way it's good to get the exercise, but I don't think I was doing it properly at the time.”* (Participant 3, woman, age 63, type 1 diabetes) *“[monitoring blood glucose levels with the CGM] is still such a difficult task as you're probably aware, of trying to maintain it within an ideal range because there are just so many things can just throw it off. Going to your [resistance] exercise or going for a walk, I'm almost certain to have a hypo because I'm not walking much in the house and then when I do exercise I'm having to make sure I've got jelly babies with me all the time* … *It all seems to be in the lap of the gods sort of thing. I mean sometimes it would work fine and then at other times you're thinking, ‘Hang on a minute why am I hypo this time?’”* Participant 6, man, age 63, type 1 diabetes
		Variable knowledge of the potential benefits of RT for diabetes	*“You see I didn't realise [until this interview] that weight training was so important.”* (Participant 6, man, age 63, type 1 diabetes)
		Lacking knowledge of how to use RT‐related equipment	*“I wouldn't be worried about my diabetes [during exercising], no. More likely be worried about not being able to work the equipment.”* (Participant 8, man, age 65, type 1 diabetes)
		***Facilitators***	
		Enablement to develop RT exercise skills	*“If we were all getting shown how to use the equipment and stuff like that, I'd be quite happy.”* (Participant 10, man, age 63, Type 2 diabetes)
			*“I wouldn't be worried about my diabetes [during exercising], no. More likely be worried about not being able to work the equipment.”* (Participant 8, man, age 65, type 1 diabetes)
Motivation	Automatic	***Barriers***	
		RT feels boring	*“No, [resistance training] wouldn't interest me at all. Personally, it would bore me to tears.”* (Participant 11, man, age 73, type 2 diabetes)
			*“I would take some convincing that lifting weights was ever going to make a significant difference over and above what other forms of exercise might*—*but if you found or showed that actually this is going to make a significant difference, then I would take it seriously. And if you could say, ‘Okay there are regimes you can follow, there's something you can do that will make it less boring and more engaging and more dare we say, fun, [then I would do them].”* (Participant 15, man, age 60, type 1 diabetes)
		Fear of injury	*“I'm slightly more frightened [about exercising] than I used to be*… *I'm frightened about my heart.”* (Participant 13, woman, age 69, type 1 diabetes)
			*“I'm sort of petrified of falling off because I'm worried something's going to break again.”* (Participant 6, man, age 63, type 1 diabetes)
		Fear of hypoglycaemia	*“Like this morning, I'd been for a walk and as I came back I thought I'm sure I'm going hypo and I looked at the reading and it said four, so I thought I can feel it so I immediately stopped my background insulin but then it still dropped. This is the trouble, they can't get the control right. I was really frightened. Very frustrating, gets me very upset.”* (Participant 13, woman, age 69, type 1 diabetes)
		***Facilitators***	
		Feeling supported by others to enable exercise	*“Just someone to check in with once a month or who would help you work out a program, motivate you to do it.”* (Participant 14, woman, age 66, type 1 diabetes)
		‘I will not let diabetes limit my choices’	*“If my wife suggests we go for a walk, then I will cheerfully go for a walk.”* (Participant 15, man, age 60, type 1 diabetes)
			*“For me, there [is] nothing I couldn't do because of diabetes.”* (Participant 1, woman, age 80, type 1 diabetes)
Motivation	Reflective	***Barriers***	
		Having other exercise routines that do not involve RT	*“I think my diabetes is better managed by walking my dogs and using my Libre. The Libre has been amazing*—*using the Libre, eating if I need to, not eating if I don't*… *so the gym would be a complicating factor now, because I guess if I went back now, I'd have to work it all out again*… *diabetes for me was an add on to my life and it was a stressful add on*… *so the gym added to that [would be] overwhelming.”* (Participant 3, woman, age 63, type 1 diabetes)
		***Facilitators***	
		Use of CGM to monitor diabetes control during exercise	*“I've always got my phone handy; if I feel a bit iffy, I can quickly scan the Libre.”* (Participant 5, woman, age 67, type 1 diabetes)
		Valuing improvements in health brought about by RT	*“Now if resistance training would help me keep strength, or regain that strength, I would most certainly do it.”* (Participant 14, woman, age 66, type 1 diabetes)
Opportunity	Social	***Barriers***	
		Feeling ‘too old’ to try a new exercise modality	*“I've never done that sort of exercise (resistance training)*…*but I don't think I'll ever start doing that sort of thing.”* (Participant 9, woman, age 89, type 1 diabetes)
			*“I'm 75, how would I go about doing anything like that [resistance training]? Am I too old?”* (Participant 7, woman, age 75, type 1 diabetes)
			*“I think I'm probably too old for your [planned resistance training RCT] study. Oh, I would never go to a gym. No, not now.”* (Participant 11, man, age 73, type 2 diabetes)
		Poor awareness in society and amongst personal trainers	*“Finding a reason to stop was how little information there was [about exercising with diabetes] and how little awareness there was.”* (Participant 3, woman, age 63, type 1 diabetes)
		Negative self‐comparison between themselves and regular gym‐goers	*“[Interviewer: Okay, was there anything about it that stopped you from going [to the gym]?] There was, and it's related to diabetes. I can't bear people treating you different because they've now discovered that you have diabetes.”* (Participant 2, man, age 60, type 1 diabetes)
			*“I do have that Arnold Schwarzenegger idea of people in the gym, and I think, ‘Oh my god, how could I go with my [Charcot foot] feet like they are and be in front of them?”* ‐Participant 1, woman, age 80, type 1 diabetes
			*“I would feel sweaty if my glucose plummeted sometimes [during exercise], I would have to just sit out in the changing room and then people think that you're just so horribly unfit you can't do it. A horrible sort of image.”* (Participant 3, woman, age 63, type 1 diabetes)
		Being encouraged to limit exercise at diagnosis	*“[At diagnosis]*—*a sort of specialist nurse said, ‘What do you do for a living?’ I said, ‘Well actually I'm qualified as a PE teacher but I'm now teaching skiing.’ She goes, ‘Well I'm sorry to say you'll never do exercise again!’”* (Participant 9, woman, age 89, type 1 diabetes)
		Partner cannot/will not accompany them to exercise	*“I would like to go possibly for more walks but as I say my husband has dreadful arthritis.”* (Participant 7, woman, age 75, type 1 diabetes)
		***Facilitators***	
		Knowledgeable personal trainers to support RT for those with insulin‐treated diabetes	*“[I would suggest to] have an empathic trainer who understood the complexity of diabetes, but also the complexity of ageing and could work out a structured program. I work better with a structure with the help of someone there who was a bit of a mentor.”* (Participant 3, woman, type 1 diabetes)
Opportunity	Physical	***Barriers***	
		Financial cost of attending a gym	*“I don't have any plans soon to start gym again. Too expensive apart from anything else.”* (Participant 14, woman, age 66, type 1 diabetes)
		Lack of time to attend a gym	*“I think that would be easier for me [to exercise at home] rather than trying to find the time to go to a gym.”* (Participant 7, woman, age 75, type 1 diabetes)
			*“Ask me to go to the gym and I would think, oh I can't fit that into my day*—*whereas I'd do it at home.”* (Participant 4, woman, age 65, type 1 diabetes)
			*“I can't stress how much impact a lack of time and ordinary life impacts the motivation to do gym training.”* (Participant 3, woman, age 63, type 1 diabetes)
		FACILITATORS	(nil)

### 3.1 Physical capability

#### 3.1.1 Barriers

##### Feeling slowed down with ageing

Ageing was frequently mentioned by participants as a factor that limited their physical capabilities, making it more difficult to engage in RT. The experience of physical decline, whether due to reduced stamina, strength, or overall energy levels, was a common theme.

It's not being able to do as much as I want to do because obviously, you're slowing down. (Participant 5, woman, age 67, type 1 diabetes)

This perceived decline in physical ability often led to a sense of frustration and resignation, as participants felt that they could no longer perform activities they once enjoyed or found beneficial.

##### Diabetes management feeling harder with age

Managing diabetes over the long term was seen to introduce additional complexities as participants aged. Increased unpredictability in blood glucose levels, variable insulin sensitivity, and overall health meant that participants viewed the incorporation of RT into their everyday lives as challenging, as they focused increasingly on managing their changing health and diabetes‐related requirements.

When you've had diabetes for so long it doesn't always follow the rules. (Participant 8, man, age 65, type 1 diabetes)

##### Specific physical limitations to training

Physical limitations were a significant barrier for many participants, including those related to diabetes complications and those related to the process of ageing. Impact of retinopathy‐ and neuropathy‐related complications, including visual impairment and foot issues, was commonly reported by participants. These limitations directly impacted their ability to engage in RT, as they struggled with balance, mobility and safety during exercises, or worried that they would experience these challenges if they tried. For some, these physical constraints were not just inconveniences but major obstacles that prevented them from participating in RT altogether. These challenges often reflected underlying frailty‐related symptoms, reinforcing participants' perceptions that RT might exceed their physical capacity.

If the shoes aren't right then that limits how I can exercise and my mobility and everything else and that has been a huge challenge… If I can't balance and stand in my shoes then doing weights for anything else is difficult for me. (Participant 1, woman, age 80, type 1 diabetes)

### 3.2 Psychological capability

#### 3.2.1 Barriers

Variable knowledge of managing diabetes, especially hypoglycaemia, during RT Participants' understanding of how to manage their diabetes during resistance training (RT) varied, with most describing limited knowledge. Some described attempts to carry out RT and manage their diabetes during training, but struggled, leading to experiences and fears of hyper‐ and hypoglycaemia. Participants described uncertainty about how to adjust their insulin or food intake before, during, or after RT.

I don't think you need to go pull weights at the gym because what that was doing for me when I was doing it as diabetic was just sending me very low and then I was correcting it and going very high… so yes in a way it's good to get the exercise, but I don't think I was doing it properly at the time. (Participant 3, woman, age 63, type 1 diabetes)

[monitoring blood glucose levels with the CGM] is still such a difficult task as you're probably aware, of trying to maintain it within an ideal range because there are just so many things can just throw it off. Going to your [resistance] exercise or going for a walk, I'm almost certain to have a hypo because I'm not walking much in the house and then when I do exercise I'm having to make sure I've got jelly babies with me all the time … It all seems to be in the lap of the gods sort of thing. I mean sometimes it would work fine and then at other times you're thinking, ‘Hang on a minute why am I hypo this time?’ (Participant 6, man, age 63, type 1 diabetes)

##### Variable knowledge of the potential benefits of RT for diabetes

Awareness of the benefits that RT could offer for diabetes management was often limited among participants, reflecting a broader issue of insufficient information dissemination about the potential positive impacts of RT on health outcomes in diabetes. When discussing exercise, most participants focused on what they perceived to be more traditional forms of exercise like aerobic activities. Several participants attributed these knowledge gaps to receiving outdated or nonspecific exercise advice earlier in their diabetes care.

You see I didn't realise [until this interview] that weight training was so important. (Participant 6, man, age 63, type 1 diabetes)

##### Lacking knowledge of how to use RT‐related equipment

Participants also expressed concerns about their ability to use RT equipment, which presented a significant barrier to engagement. The unfamiliarity with gym equipment—whether due to a lack of prior exposure or insufficient instruction—made the prospect of starting or continuing RT daunting for many. This lack of confidence in using equipment created a psychological barrier, preventing individuals from fully engaging in RT, even when they might be motivated to do so.

I wouldn't be worried about my diabetes [during exercising], no. More likely be worried about not being able to work the equipment. (Participant 8, man, age 65, type 1 diabetes)

### 3.3 Psychological capability

#### 3.3.1 Facilitators

##### Enablement to develop RT exercise skills

Participants noted that being supported in developing the necessary skills to safely engage in RT would help overcome some of their concerns about the exercise. Many felt that structured guidance in using RT equipment would build their confidence and willingness to participate.

If we were all getting shown how to use the equipment and stuff like that, I'd be quite happy. (Participant 10, man, age 63, Type 2 diabetes)

### 3.4 Automatic motivation

#### 3.4.1 Barriers

##### RT feels boring

Participants often described RT as monotonous and unengaging, even if they had little to no experience with it, which significantly deterred them from participating

No, [resistance training] wouldn't interest me at all. Personally, it would bore me to tears. Participant 11, man, age 73, type 2 diabetes

This perceived monotony was particularly challenging for those who were used to more aerobic forms of exercise or who required a higher level of stimulation to stay engaged. This perception was, for some, based on limited exposure to RT, and an acknowledgement that structured guidance or variation might make it more engaging.

I would take some convincing that lifting weights was ever going to make a significant difference over and above what other forms of exercise might—but if you found or showed that actually this is going to make a significant difference, then I would take it seriously. And if you could say, ‘Okay there are regimes you can follow, there's something you can do that will make it less boring and more engaging and more dare we say, fun, [then I would do them]. (Participant 15, man, age 60, type 1 diabetes)

##### Fear of injury

Fear of injury was a significant deterrent for many participants. This fear was particularly prevalent among those with diabetes‐related complications or an unrelated history of injury, leading to anxiety about the potential risks associated with RT. Participants worried that the physical demands of RT could exacerbate their health conditions or lead to new injuries, making them hesitant to participate.

I'm slightly more frightened [about exercising] than I used to be… I'm frightened about my heart. (Participant 13, woman, age 69, type 1 diabetes)

##### Fear of hypoglycaemia

Fear of hypoglycaemia was also a distinct, significant deterrent to RT expressed by participants. This was primarily shaped by their prior experiences of glucose instability during or after physical activity. For some, the unpredictability of blood glucose responses created a sense that exercise carried immediate personal risk, even when the activity itself might otherwise have felt manageable.

Like this morning, I'd been for a walk and as I came back I thought I'm sure I'm going hypo and I looked at the reading and it said four, so I thought I can feel it so I immediately stopped my background insulin but then it still dropped. This is the trouble, they can't get the control right. I was really frightened. Very frustrating, gets me very upset. (Participant 13, woman, age 69, type 1 diabetes)

### 3.5 Automatic motivation

#### 3.5.1 Facilitators

##### Feeling supported by others to enable exercise

Social support was identified as a key facilitator to exercise, with participants indicating that encouragement from others, including partners or personal trainers, would motivate them to engage in RT. The presence of someone to check in or to help design an exercise programme was seen as particularly beneficial.

Just someone to check in with once a month or who would help you work out a program, motivate you to do it. (Participant 14, woman, age 66, type 1 diabetes)

##### ‘I will not let diabetes limit my choices’

Participants who felt a strong internal drive to not let diabetes control their choices saw this as a significant motivator to engage in RT. For them, maintaining independence and control over their activities was essential.

For me, there [is] nothing I couldn't do because of diabetes. (Participant 1, woman, age 80, type 1 diabetes)

### 3.6 Reflective motivation

#### 3.6.1 Barriers

##### Having other exercise routines that do not involve RT

Several participants preferred other forms of exercise over RT due to their established routines and the perceived complexity of incorporating RT into their lives. For some, more familiar activities like walking offered sufficient management of their diabetes without the need for additional complexity or new learning that RT was perceived to bring. This preference for simpler, more integrated forms of exercise reflects a desire to avoid the added mental and physical demands that RT might entail. Participants frequently described the anticipated effort of re‐learning glucose management for a new activity as a major disincentive to adopting RT.

I think my diabetes is better managed by walking my dogs and using my Libre. The Libre has been amazing—using the Libre, eating if I need to, not eating if I don't… so the gym would be a complicating factor now, because I guess if I went back now, I'd have to work it all out again… diabetes for me was an add on to my life and it was a stressful add on… so the gym added to that [would be] overwhelming. (Participant 3, woman, age 63, type 1 diabetes)

### 3.7 Reflective motivation

#### 3.7.1 Facilitators

##### Use of CGM to monitor diabetes control during exercise

The use of continuous glucose monitoring (CGM) was identified as a facilitator by participants, as it gave them confidence in managing their blood glucose levels during RT. Knowing they could monitor their condition in close to real‐time reduced anxiety related to hypoglycaemia during exercise.

I've always got my phone handy; if I feel a bit iffy, I can quickly scan the Libre. (Participant 5, woman, age 67, type 1 diabetes)

##### Valuing improvements in health brought about by RT

Despite some initial resistance, some participants recognised the potential health benefits of RT, particularly in maintaining or regaining strength. This acknowledgment of RT's positive impact on physical health suggests that, when participants are aware of the benefits and they become more tangibly related to their diabetes control and overall health, they may be more motivated to engage in RT. For these individuals, the improvements in strength, mobility and overall health that come with regular RT were seen as valuable enough to overcome any initial barriers.

Now if resistance training would help me keep strength, or regain that strength, I would most certainly do it. (Participant 14, woman, age 66, type 1 diabetes)

### 3.8 Social opportunity

#### 3.8.1 Barriers

##### Feeling ‘too old’ to try a new exercise modality

The perception of being “too old” to start a new form of exercise like RT was a significant barrier for many participants. This belief was especially prevalent among older adults who had never engaged in RT before and who felt that it was not appropriate or feasible to begin at their age.

I'm 75, how would I go about doing anything like that [resistance training]? Am I too old? (Participant 7, woman, age 75, type 1 diabetes)

I think I'm probably too old for your [planned resistance training RCT] study. Oh, I would never go to a gym. No, not now. (Participant 11, man, age 73, type 2 diabetes)

##### Poor awareness in society and amongst personal trainers of how to manage diabetes with RT

A significant barrier identified was the lack of awareness and knowledge among personal trainers and the broader society about how to manage diabetes in the context of RT. Participants expressed frustration at the limited understanding of their condition within the fitness industry, which often led to feelings of alienation or being misunderstood. They felt that they needed to receive appropriate support and guidance to take part in RT safely.

Finding a reason to stop was how little information there was [about exercising with diabetes] and how little awareness there was. (Participant 3, woman, age 63, type 1 diabetes)

##### Negative self‐comparison between themselves and regular gym goers

Participants frequently compared themselves unfavourably to regular gym goers, which often deterred them from participating in RT. This self‐comparison was exacerbated by the physical manifestations of their diabetes, such as visible complications or the need for frequent breaks, which made them feel out of place in a typical gym environment.

[Interviewer: Okay, was there anything about it that stopped you from going [to the gym]?] There was, and it's related to diabetes. I can't bear people treating you different because they've now discovered that you have diabetes. (Participant 2, man, age 60, type 1 diabetes)

I do have that Arnold Schwarzenegger idea of people in the gym, and I think, ‘Oh my god, how could I go with my [Charcot foot] feet like they are and be in front of them? (Participant 1, woman, age 80, type 1 diabetes)

##### Being encouraged to limit exercise at diagnosis

Several participants described their experiences where they were discouraged from engaging in exercise, including RT, at the time of their diabetes diagnosis. This early discouragement, often delivered by healthcare professionals, had long‐lasting effects, creating a significant psychological barrier to exercise that persisted even as their condition evolved. These early messages often shaped long‐term beliefs about what was safe or appropriate, even decades later.

[At diagnosis]—a sort of specialist nurse said, ‘What do you do for a living?’ I said, ‘Well actually I'm qualified as a PE teacher but I'm now teaching skiing.’ She goes, ‘Well I'm sorry to say you'll never do exercise again!’ (Participant 9, woman, age 89, type 1 diabetes)

##### Partner cannot/will not accompany them to exercise

For some participants, the inability of their partner to join them in exercise activities due to their own health issues was a barrier. The social aspect of exercising together was an important motivator for many.

I would like to go possibly for more walks but as I say my husband has dreadful arthritis. (Participant 7, woman, age 75, type 1 diabetes)

### 3.9 Social opportunity

#### 3.9.1 Facilitators

##### Knowledgeable personal trainers to support RT

Participants highlighted the importance of having a personal trainer who understands both diabetes and ageing. They felt that such trainers could offer tailored support, providing structured exercise programmes that account for their specific health needs and limitations. They felt that this would help them feel more confident to engage with RT and reduce concerns about managing their diabetes or injury risk.

[I would suggest to] have an empathic trainer who understood the complexity of diabetes, but also the complexity of ageing and could work out a structured program. I work better with a structure with the help of someone there who was a bit of a mentor. (Participant 3, woman, type 1 diabetes)

### 3.10 Physical opportunity

#### 3.10.1 Barriers

##### Financial cost of attending a gym

The financial cost associated with gym memberships was a significant barrier for several participants, limiting their perceived ability to engage in RT, as they viewed RT as only achievable in a traditional gym environment. The expense of gym access, particularly for those on pension income, made it difficult to justify the cost of a membership, even when they were motivated to participate in RT.

I don't have any plans soon to start gym again. Too expensive apart from anything else. (Participant 14, woman, age 66, type 1 diabetes)

##### Lack of time to attend a gym

Time constraints were frequently mentioned as a barrier to attending a gym. Despite most participants being retired from full time work, they expressed that they were still busy with various daily commitments which often left little time for exercise, making it challenging to maintain a regular RT routine. Many identified that they would consider carrying out RT at home as they could fit it into their existing commitments.

Ask me to go to the gym and I would think, oh I can't fit that into my day—whereas I'd do it at home. (Participant 4, woman, age 65, type 1 diabetes)

I can't stress how much impact a lack of time and ordinary life impacts the motivation to do gym training. (Participant 3, woman, age 63, type 1 diabetes)

## 4 DISCUSSION

This study highlights significant barriers and facilitators related to engaging in resistance training (RT) among older adults living with insulin‐treated diabetes and prefrailty. Using the COM‐B model, we identified significant behavioural influences to RT across several domains. Barriers to RT centred particularly on those related to psychological and physical capability, and social opportunity. We also identified that awareness of the benefits of RT specifically on diabetes management and frailty‐related health issues was poor, as were the self‐reported skills required to carry it out. Most participants felt physically unable to try to carry out this modality of exercise due to their health and frailty‐related symptoms, building on a history of difficulty exercising with diabetes when younger and often being warned off exercise by clinical staff at diagnosis. Facilitators to RT were limited and fell primarily into psychological capability, reflective motivation and social opportunity domains. Participants felt that being supported to carry out RT in terms of skill development, having a supportive partner, and a diabetes‐aware RT coach, would build on any existing awareness of the benefits of RT for their health and ultimately help them to perform RT. In terms of frailty, whilst eligibility was defined using a clinical frailty scale, participants rarely described themselves as living with prefrailty, and frailty status was not discussed in their accounts of exercise decision making. Instead, perceptions of RT appeared to be shaped by functional concerns associated with ageing and diabetes complications, such as feeling slowed down, fear of injury and reduced confidence. This suggests that intervention messaging may be more acceptable when framed around maintaining strength, function and independence, rather than explicitly focusing on frailty.

These barriers reflect those found in similar populations without diabetes, particularly around limited physical capability (e.g. frailty, pain), time constraints and inadequate support for exercise engagement.^24^, ^25^, ^26^ Fear of hypoglycaemia and injury, especially among older adults using insulin, has also been well documented as a reason for avoiding physical activity.^24^, ^27^ Additionally, co‐morbidities such as neuropathy and cardiovascular issues, which were frequently mentioned by participants in this study, are recognised as barriers to physical activity among older adults with diabetes.^28^, ^29^

Currently, there is limited information on the most effective RT protocols specifically tailored for the needs of those with insulin‐treated diabetes,^30^ especially for those who are older and living with prefrailty. The current study identifies not only the barriers, but also the facilitators to RT engagement in this population, which can inform the design of more acceptable and tailored RT programs and associated holistic support. For instance, (a) targeted education on the benefits of RT for managing diabetes and frailty, (b) personalised RT programmes using trainers who understand the unique needs of older adults with diabetes, (c) using continuous glucose monitoring (CGM) to help participants feel more confident about managing their glycaemic levels during RT, reducing the fear of hypoglycaemia, (d) providing structured skill development sessions to teach participants how to use RT equipment safely and effectively, and (e) developing social support networks, such as group RT sessions or family involvement, to maintain motivation and reduce feelings of isolation.

This is the first study to focus on a specific and vulnerable population, and to apply a well‐evidenced behavioural science framework (COM‐B) to analyse data and identify explanatory factors related to engagement in RT. We specifically included non‐obese, insulin‐treated older adults with type 2 diabetes based on clinical observations, particularly in secondary care, that at this stage some individuals share features with older adults with type 1 diabetes, including mild frailty, hypoglycaemia risk, insulin management challenges, and the fact that further weight loss may be undesirable. However, recruitment of this relatively smaller subgroup proved challenging. As a result, the sample was weighted towards type 1 diabetes, and the findings should be interpreted primarily as reflecting the experiences of older adults with type 1 diabetes. Given the marked pathophysiological differences between type 1 and type 2 diabetes, these groups should not be assumed to be interchangeable, and the present findings should therefore be interpreted with particular caution in relation to older adults with insulin‐treated type 2 diabetes. The small number of participants with type 2 diabetes also precluded meaningful comparison between diabetes types, although these clinical overlaps suggest that there may be some convergence in experiences at this stage of disease. Future work would benefit from purposive sampling and, where feasible, separate analysis of older adults with type 1 diabetes and those with insulin‐treated type 2 diabetes to examine where experiences converge and where they differ.

This study highlights that knowledge gaps and perceptions pose significant barriers to RT and exercise in this population, but also that these challenges can be addressed. A collaborative approach between diabetes clinics and public health services could develop an RT support package tailored for this group, similar to the ‘exercise prescriptions’ successfully used in areas like cardiac rehabilitation. Informed by our COM‐B findings, future RT programmes for this group should include structured education on RT‐specific glucose management, and explicit reassurance about safety in the context of long‐standing diabetes complications. Programmes should provide graded, individualised progressions with supervised instruction to build basic equipment skills and reduce fear of injury, alongside accessible delivery options (for example, home‐based or community provision) to minimise cost and time barriers. Embedding social support (for example, exercise partners, small groups, or trainer check‐ins) and ensuring staff are trained in diabetes‐specific considerations may further improve confidence and long‐term adherence.

## AUTHOR CONTRIBUTIONS

DJW and RS secured funding for this work and designed the study. SR, GST, MDW and JAMS contributed to study design, reviewed, and edited the manuscript. RS collected all data. RS, SR and DJW analysed data. All authors reviewed and approved the final version of the manuscript. RS is the guarantor of this work and, as such, had full access to all the data in the study and takes responsibility for the integrity of the data and the accuracy of the data analysis.

## FUNDING INFORMATION

This study was supported by a Wellcome Trust ISSF award to DW and RS, and the Francis James Bell Endowment, County Durham Community Foundation, award to DW. JAMS' research is supported by the Ray Wilson Memorial Fund. MDW acknowledges support from the NIHR Newcastle Biomedical Research Centre, the NIHR Newcastle Clinical Research Facility and the Multiple Long‐term Conditions Cross‐NIHR Collaboration.

## CONFLICT OF INTEREST STATEMENT

The authors declare no conflicts of interest.

## PRIOR PRESENTATION

An early version of this work was presented at the 2023 ADA conference, San Diego, USA, June 23–26.
